# Setting and meeting priorities in Indigenous health research in Australia and its application in the Cooperative Research Centre for Aboriginal Health

**DOI:** 10.1186/1478-4505-7-25

**Published:** 2009-11-20

**Authors:** Johanna M Monk, Kevin G Rowley, Ian PS Anderson

**Affiliations:** 1 Onemda VicHealth Koori Health Unit, Centre for Health and Society, Melbourne School of Population Health, University of Melbourne, Victoria 3010, Australia

## Abstract

Priority setting is about making decisions. Key issues faced during priority setting processes include identifying *who *makes these decisions, *who *sets the criteria, and *who *benefits. The paper reviews the literature and history around priority setting in research, particularly in Aboriginal health research. We explore these issues through a case study of the Cooperative Research Centre for Aboriginal Health (CRCAH)'s experience in setting and meeting priorities.

Historically, researchers have made decisions about what research gets done. Pressures of growing competition for research funds and an increased public interest in research have led to demands that appropriate consultation with stakeholders is conducted and that research is of benefit to the wider society. Within Australian Aboriginal communities, these demands extend to Aboriginal control of research to ensure that Aboriginal priorities are met.

In response to these demands, research priorities are usually agreed in consultation with stakeholders at an institutional level and researchers are asked to develop relevant proposals at a project level. The CRCAH's experience in funding rounds was that scientific merit was given more weight than stakeholders' priorities and did not necessarily result in research that met these priorities. After reviewing these processes in 2004, the CRCAH identified a new facilitated development approach. In this revised approach, the setting of institutional priorities is integrated with the development of projects in a way that ensures the research reflects stakeholder priorities.

This process puts emphasis on identifying projects that reflect priorities prior to developing the quality of the research, rather than assessing the relevance to priorities and quality concurrently. Part of the CRCAH approach is the employment of Program Managers who ensure that stakeholder priorities are met in the development of research projects. This has enabled researchers and stakeholders to come together to collaboratively develop priority-driven research. Involvement by both groups in project development has been found to be essential in making decisions that will lead to robust and useful research.

## Introduction

Priority setting is about making decisions and planning to make sure we use limited resources well. Identifying a research agenda, and the means by which it is identified, is a strategic process to ensure that organisations are transparent and accountable in the expenditure of funds; that stakeholders will be involved in decision-making; and that research will be useful. While there has been much discussion since the 1980s on how organisations decide to strategically allocate limited funding to research, much of the implementation of priority setting in health research did not begin until the 1990s, as we shall discuss in this paper.

Indigenous communities--particularly in settler nations such as Australia, New Zealand and Canada--have demanded involvement in priority setting processes. Since colonisation, these communities have experienced poor health in comparison to non-Indigenous citizens of their nations, and research has not contributed to effectively addressing this inequity. The urgent need to improve Indigenous health and redress the power imbalance between the researcher and the researched has resulted in challenges to traditional research, which we will discuss in this paper. While investigator-driven research has been seen by Indigenous communities to benefit researchers rather than communities, it is through reforming the institutional structures that changes can be made to the research being done at a project level.

In this paper we will explore the literature and history around priority setting in research and look at the experience of the Cooperative Research Centre for Aboriginal Health (CRCAH) in developing a system for setting research priorities with Australian Aboriginal people. This system involves listening to stakeholders' advice on what research will be useful to them, and facilitating the development of the projects with researchers to ensure that these needs are addressed.

## Background

### History of priority setting

To understand how the concept of priority setting has influenced the research process, we first need to understand how research topics have traditionally been chosen. From a position of primarily investigator-initiated research in the prosperous period following World War II, recent, economic restrictions have led to a greater focus on research addressing specific priorities, as reflected in Don Aitken's analysis of trends in funding in the Australian Research Council (ARC) and the Australia Science and Technology Council (ASTEC) from the early 1970s to 1990s.

Aitken recalls that in the 1970s there was no clear sense of how to allocate money other than historically and that the holy writ was that no person shall tell another what to work on. Researchers have argued (accurately) that research discoveries were not always predictable, accidental findings happened and what might be an uninteresting discovery in one field might have immense importance in another. The freedom of researchers was preached by those who believed that excellence must rule, no matter what national needs were, and it was assumed that other people would use the research results to develop products, processes or policies [1].

When funding bodies allocated monies for research, 'peer review' - or assessment by other researchers - has been internationally accepted as the main form of decision-making [2-4]. However, the literature on peer review processes has identified weaknesses such as conflict of interest, lack of transparency, and reinforcing of power structures [2,3]. Although some still argue that assessment of excellence or quality by other researchers is the most appropriate method for judging research proposals [5], researchers are no longer unquestioningly accepted as the only group to decide on which research should be funded.

The belief in academic freedom and peer review has been used to justify the tradition of using excellence as a basis for funding research. Priority setting initiatives challenged these traditions and consequently were initially met with caution and skepticism by many researchers [1,6,7]. Below we will discuss why researchers, such as Aitken, and other stakeholders are increasingly supporting priority setting processes in research funding. We will particularly discuss these changes in Aboriginal health where the benefits of research for Aboriginal people have traditionally been limited or absent.

### Research impact

A body of literature has recently been developed around the use or impact of research. It addresses the questions about how public money that is invested in research can respond to the problems facing society [8,1]. Imperative in this discussion is the issue of how we prioritise research to be funded.

Internationally, health research priority setting processes aim to produce research that will have wider benefits to our society. These benefits might arise from research being used in developing policy [9], in improving health systems [10] or the contribution of research towards improving health outcomes [11]. Many organizations have begun to discuss how their research could make such impacts. An example of this can be seen in the Australian National Health and Medical Research Council's (NHMRC) review in the late 1990s.

In 1998, the NHMRC produced a discussion document based on consultation conducted as part of the Health and Medical Strategic Review, chaired by Peter Wills. *The Virtuous Cycle*, (commonly known as the Wills Report) found that there was an urgent need for more priority-driven research that would contribute directly to the health of the population and an evidence based health system, albeit alongside fundamental research which might produce research that is high impact and innovative. The imperative for more priority-driven research came from the recognition that ' [r]esearch is an important direct contributor to improving the health of the Australian population and increasing effectiveness, efficiency and equity in a health system coming under ever-increasing pressure' [4].

### Transparency and Accountability

The growing awareness of the impact of research is one of the factors that has led to an increased demand from the general community for more transparency and accountability in determining how research resources are allocated. As discussed above, researchers claimed they had a right to determine their own agenda, but it could not be assumed that researchers were immune to external influences and cultural biases. Tension has arisen between researcher autonomy and the relevance of research to the public who fund it through their taxpayer dollar [1,3,10].

A significant increase in research activity and the number of researchers has had consequences for funding. Agencies must make difficult decisions when allocating funds and, consequently, 'greater numbers of applications that deserve to be supported are being turned down' [5]. For example in 2003 in Australia, the NHMRC received 1798 applications and funded 407, or 23 per cent of these [12]. Priority setting provides a rational process for allocating health research funding, because the available funds are low in comparison to the very high potential benefits [13].

Tight budgets have given weight to the argument for transparent processes [1]. It is not enough for funding agencies to design an appropriate system for making decisions about allocating funding. While there are still some who are prepared to accept a messy and imprecise process if the results are good, there is pressure on agencies to explain and justify the methods for setting priorities from those who expect practical and visible results [11]. They want to make sure the research that is funded will have the maximum impact on society.

### Linking researchers and stakeholders

A transparent process for determining priorities must also be open about whose interests are represented. The identification of priorities will be influenced by the stakeholders who participate in the process [10,13].

Priority setting is no longer just the domain of researchers. The range of individuals and organisations who claim a right to be involved in decision-making has grown substantially over the past few decades [14]. In addition to researchers, a multitude of research users, including policy-makers, managers, practitioners, service users and lobby groups, are involved in priority setting exercises described in the literature.

A useful example of how different stakeholders can affect the priorities identified is given by Sally Redman and colleagues in a paper about a priority setting process conducted by the NHMRC National Breast Cancer Centre in 1995. Consultation workshops were held in each state and territory. It was decided that in addition to these regional workshops, extra workshops would be held for Aboriginal and Torres Strait Islander people, those from a non-English speaking background and rural and remote residents. These targeted workshops identified different priorities to those of the general public. For example the issue of culturally acceptable health care for those with breast cancer was a high priority for Aboriginal and Torres Strait Islander people but did not emerge as a priority in the regional workshops [15].

As this example demonstrates, some groups will dominate priority setting and minority voices will not necessarily be heard. The literature cautions that, when choosing participants, it is important to ensure an appropriate balance between public and expert input [9,10,16]. While the input of researchers is acknowledged to be important, it is of concern that there is a danger of them being overrepresented in the priority setting processes and particular attention must be given to encouraging other stakeholder groups to participate [10].

Priority setting has been described as a debate [10], a consensus building process [17] and a political process [18]. Ensuring a range of voices in priority setting is considered important because, as well as identifying useful research, there are the additional benefits from bringing together researchers and other stakeholders. Jonathon Lomas and colleagues argue that this promotes 'linkage and exchange' [10]. The experiences of the CRCAH, as described below, support this view.

Exchanges of views can contribute towards the development of effective partnerships between stakeholders and researchers. For example the Australian Primary Health Care Research Evaluation and Development priority setting process 'allowed debate about roles, responsibilities and understanding, as well as identifying and making explicit the tensions between the values, needs and perspectives of different stakeholder groups' [19]. As these groups will often work together in carrying out and implementing the research, it is important to nurture their relationship.

Linking these groups can also increase their commitment to the research. Health system managers and policy makers are more likely to implement changes based on the results of the research [10] and researchers will be more committed to doing research identified as a priority [19]. Hence, participation by a range of groups in the priority setting process can strengthen the research and increase the likelihood of it being used.

### Priority setting in Aboriginal health research

Who makes decisions about priorities for research has long been a source of concern for Aboriginal communities. As the above example of the NHMRC National Breast Cancer Centre illustrates, the priorities of Aboriginal people are not necessarily the same as that of the general population. In the 1980s, Aboriginal people began to demand Aboriginal control over research development and funding [20]. The *National Aboriginal Health Strategy *in 1989 called for Australian Aboriginal people to define the problems, rather than research reflecting the fancy of the researcher [21].

Internationally, Indigenous people have called for change to the way research is done. By the mid 1990s, Indigenous people were speaking out about their perception of non-Indigenous researchers making decisions about research agendas or what to research [20,22]. They viewed research as contributing to the colonisation process by benefiting researchers rather than communities. But they also identified the potential to contribute to self determination processes and improved health through community controlled research [2,23-27].

In a discussion of the history of the development of Aboriginal research in Australia, Kim Humphery noted that the reform of Indigenous health research has primarily focused on 'the individual act of research, on the particular project, as *the *site for transforming research practice.' He called for changes not just to research practice but also to ways of identifying, funding, and controlling research in order to bring about a transformation 'involving shifts in *institutional *arrangements' [20]. That is, a change was required in the ecology of the research landscape.

By the end of the 1990s, two organisations were considering setting priorities in Australian Aboriginal health research at an institutional level--the NHMRC and the Cooperative Research Centre for Aboriginal and Tropical Health (CRCATH). Both organisations were responsible for funding a considerable amount of Aboriginal health research in Australia, and their review of processes would have a great impact on setting priorities for research with Australian Aboriginal communities.

The NHMRC is 'Australia's peak body for supporting health and medical research; for developing health advice for the Australian community, health professionals and governments; and for providing advice on ethical behaviour in health care and in the conduct of health and medical research' [28]. It was established under the *National Health and Medical Research Council Act 1992 *and its Chief Executive Officer reports directly to the Commonwealth Government's Minister for Health and Ageing.

In 1997, the NHMRC and the Office for Aboriginal and Torres Strait Islander Health (in the Commonwealth Government's Department of Health and Ageing) established the Aboriginal and Torres Strait Islander Research Agenda Working Group (RAWG) as part of their commitment to developing a coherent and coordinated approach to addressing the health research needs of Aboriginal and Torres Strait Islander peoples [29]. The Wills Report highlighted that Indigenous people were suffering a higher burden of illness than non-Indigenous Australians and recommended that strategic research be funded in under-researched areas, such as Aboriginal health [4]. In 2001, the RAWG began the development of a road map to identify national research priorities in Aboriginal and Torres Strait Islander health.

Consultation workshops brought together representatives of Aboriginal communities and organisations, academic or research organisations, governments, and research funding agencies to have input into setting priorities for research. Participants described research in the past 'as being too focussed on the priorities and career objectives of researchers rather than the priorities of communities, as at times irrelevant and non-inclusive, and as not contributing sufficiently to health improvements'. They supported partnerships between researchers and communities that built on Aboriginal self-determination and cultural respect. Identifying community-driven priorities was regarded as an important part of the research process, but time and resources were required from research funding agencies and research partners to develop such processes [29].

In 2008 the NHMRC undertook a review of the road map and found it to have contributed towards a higher quality of Indigenous research through promoting the increased participation of Indigenous people in research and an increased valuing of Indigenous methodologies. However limitations in its use were partly attributed to 'difficulties experienced by researchers in negotiating a 'traditional' grant-making process for work which requires active participation of Aboriginal and Torres Strait Islander communities and researchers' [30].

The second institutional setting where priorities for Aboriginal health research were strategically identified was the Australian Government's Cooperative Research Centre (CRC) program. From 1997 to 2003, the CRC program funded the Cooperative Research Centre for Aboriginal and Tropical Health (CRCATH), followed by a second CRC, the Cooperative Research Centre for Aboriginal Health (CRCAH), which was funded from 2003 to 2010. We will focus on this latter program, but in order to do so, the work done by the CRCATH must be considered because it made an important contribution to the development of the CRCAH's priority setting processes.

### The CRCATH - predecessor to the CRCAH

The CRCATH was established under the Cooperative Research Centres Programme to bring together researchers and research users to improve Aboriginal Health. The Cooperative Research Centres Programme was established in 1990 to create links between researchers and industry to collaborate on research that can be readily used and commercialised. The CRCATH and CRCAH were public good CRCs, which aimed to produce social benefits rather than developing commercial products. Their 'industry' partners were health providers and government departments who could apply the research results in their policies and programs.

In 2003, John Henry wrote a report on the Cooperative Research Centres for Aboriginal Health that gives a useful overview of the life of the CRCATH and the establishment of the CRCAH. He discusses the difficulties in bringing together what the CRC program called 'industry' partners (Aboriginal medical services and government departments) and 'researcher' partners (universities and research institutes), whom he describes as 'initially suspicious and cautious bedfellows'. Years of negotiation amid hostility and mistrust preceded the commencement of the CRCATH in 1997. An important element of the structure of the CRCATH was the independent Aboriginal chairperson and Aboriginal majority Board of Management, both of whom were able to exert influence over the research program [31].

As well as trying to ensure the right research was being undertaken by its researchers, the CRCATH focused on exploring processes for doing research in a way that was acceptable to Aboriginal stakeholders. One strategy was the *Action Research into Managing, Undertaking and Disseminating Aboriginal Health Research for Improved Health Outcomes Project*, which became known as the Links Project. This research resulted in the development of the Indigenous Research Reform Agenda (IRRA), which outlined the principles to be adhered to by researchers in the CRCATH. This was in line with the CRC programme's focus on capacity development (educating and training more researchers in Indigenous health) and research transfer (making sure the research was useful).

The Links Project produced six monographs that gave an overview of the literature on Indigenous health research. The third monograph (*Changing Institutions*) found that ' [w]ithin the Indigenous health research field, priority driven research is supported by those who contend that the historical prevalence of 'investigator-driven' research has resulted in insubstantial gains when measured in terms of improvements to Indigenous health outcomes' [25]. Henry found that 'the struggle to move away from a researcher-initiated approach to a more priority-driven approach has required a change in the research culture of the core research-oriented partners of the CRCATH.' In concluding his study of the CRCs for Aboriginal Health, he found that the CRCATH facilitated the utilisation of research by providing an independent domain in which its partners 'could engage with each other, work together and get to know and understand each other better. The improved relationships that were able to be developed amongst health professionals within the space provided by the CRCs flowed into the conduct of research, policy-making and service implementation'.

The benefits of bringing together CRCATH partners were reflected in the development of the CRCAH and its priority setting processes. The CRCAH had doubled the number of partners and expanded the geographic scope of the CRCATH. In April 2003, months before the commencement of the CRCAH, a meeting of the new community of stakeholders provided an opportunity to start planning the research agenda, to develop shared understandings, and to enable representatives to meet Board members and each other [31].

### Setting up the CRCAH

The literature review, which was carried out as part of the Links Project, reflected on Aboriginal health research as part of the problem associated with Indigenous disadvantage and marginalisation. It called for research 'to impact positively on the achievement of improved health outcomes for Indigenous peoples'. The authors declared that institutions that wish to support a priority-driven approach must reform their funding processes. Institutions should take responsibility for development and ongoing processes for evaluation, methodological development, community consultation and participation, ethical approval, professional development, and dissemination of research findings [25].

The history of the CRCAH could be seen as ongoing development, reflection and refinement of processes to reform Aboriginal health research in order to produce research that contributes to improvements in Aboriginal health. The planning processes that led to the establishment of the CRCAH involved identification of a broad research agenda, but decisions about how to allocate funding were yet to be made. The experience of identifying, developing, and approving research projects illustrates the challenges and rewards of this process of reform.

In 2002 the CRCAH Business Plan identified four overlapping Research Themes, which were 'outcomes of a lengthy consultation and planning process involving partners'. Theme Leaders were appointed to provide expert advice and oversee the development of projects within each Research Theme. These Leaders were chosen to represent both researcher and research user organisations.

The appointment of Theme Leaders was one of the mechanisms put in place to achieve the CRCAH's objective that research users play a full, if not dominant, role in priority setting processes. Other mechanisms for priority setting were initially through participation in the annual meetings, or Convocations, and through representation on the Aboriginal-majority Board of Management. Additionally, Aboriginal medical services and other stakeholders would be facilitated to engage with the CRCAH through a Small-to-Medium Enterprise (SME) forum, as required under CRC Program funding criteria.

The four Research Themes were the subjects of much discussion. Representatives of partner organisations met in small groups to consider them at the mini-Convocation in April 2003 and at the first full Convocation of the CRCAH in November 2003. The Research Development Group (RDG), comprising the Research Director and Theme Leaders, sought a focus that might translate into research funding. Taking these discussions into account, in early 2004 the Board identified nine emerging priorities [32].

### The first grant funding round

The CRCAH's first research commissioning round commenced in March 2004 with a call for expressions of interest based on three of the research priority areas and one open category for research topics proposed by partners. A grant assessment process was undertaken over the remainder of 2004. It was similar to NHMRC processes and involved 33 expressions of interest that were whittled down to nine full proposals. These were assessed by four research assessment panels, 25 reviewers, the RDG, and the Board. Three projects were funded out of that commissioning round. One appeal against the decision was received and dealt with.

The grant assessment and funding process was generally considered less than satisfactory. The *Practical Solutions for Peer Review *project, undertaken by Street and colleagues, involved interviewing key players in this commissioning process and a competitive system of grant funding was seen by many stakeholders to be divisive. A need for a system that better supported collaboration was identified [33]. The project report described the process of competitive peer reviewing as 'bruising to those subjected to criticism' and not conducive to encouraging and developing new Indigenous researchers [2].

Although the CRCAH made a commitment to supporting researchers and particularly Indigenous researchers, the report by Street and colleagues suggested this did not necessarily happen in practice. Time constraints and participants' confusion around processes, as well as limited capacity of the CRCAH, were seen to be reasons for this breakdown of processes. Theme Leaders were not employed by the CRCAH and often had full time employment in partner organisations. The expectation that they could mentor researchers proved to be unrealistic, given their other commitments, geographic constraints and lack of an environment to support mentoring. Some of the feedback suggested that they felt unsupported and open to criticism that was personal, rather than professional. One Theme Leader reported that 'I'm not sure how valuable my feedback was to those who did make contact with me. I did my best in the time available' [2].

Thus, the conventional model of research funding was not found to meet the needs of stakeholders [33]. Although community stakeholders (in their roles as Convocation attendees, Board members, and Theme Leaders) were involved at each step of setting the priorities, it seems that the structures of the CRCAH did not support them to translate their priorities into research projects. Some of the community-driven proposals, despite being initiated and supported by the Aboriginal health industry, were not considered for funding because they needed further development before they could meet the technical criteria.

There was a perception that industry partners were not being involved to the extent envisaged in the CRCAH Business Plan. One industry interviewee remarked that ' [w]e can't and shouldn't have to compete with university departments'. Interviewees expressed considerable concern about the degree of industry and community involvement in decision making. They identified the need for the process to be more developmental and to support community members to participate on an equal footing with university researchers [2].

In reflecting, towards the end of 2004, on its research commissioning processes, the CRCAH described them as 'cumbersome and competitive, causing fractures within what must be a collaborative organization' [34]. It was clear to many in the CRCAH that changes were required. Drawing on stakeholder feedback, the nine emerging priorities of March 2004 were reduced to a more manageable five program areas. But this time, the approach to allocating funding was drastically revised. No one wanted to go through a traditional grant-funding round again.

### The programmatic approach

Five program areas were articulated in *An Integrated Programmatic Approach to the CRCAH's Research and Development Activities, October 2004*. This document outlined the new approach and reflected on the areas for improvement. Some of the key issues to be addressed were: most projects had been initiated by researchers; projects had not often arisen from specific industry needs; and Indigenous organisations had found it difficult to put forward projects. The new approach was described as follows:

The proposed programmatic approach will see a focus on specific outcomes as identified by industry... [R]esearch projects will be brought together into a coherent program to explore what might be achieved from this base of work, what important gaps in knowledge exist, how research findings can be transferred and knowledge shared, and how capacity can be built around each area [34].

The five program areas were broad and it was agreed that industry input and collaboration was essential in identifying priorities for allocating funding to specific activities within each program. Program Leaders were appointed to perform similar overseeing roles to Theme Leaders. A major change, however, was that these Leaders were supported by Program Managers who were employed by the CRCAH. Participants in the grant funding round had stressed the importance of CRCAH involvement in the development of proposals to broker collaboration between communities, service providers, and academic researchers [33].

Program Managers were chosen for skills in facilitation and negotiation, as well as their ability to take responsibility for operationalising the programs. They were to be available to support both Program Leaders and the participants in the development of research. In particular, they were employed to make it as easy as possible for research users to participate in the research. This was achieved through brokering relationships between researchers and research users [35]. The change in organisational structure helped shift the control of the research process from researchers to research users.

### The facilitated development process

Reflecting on the grant-funding round in its 2005/2006 Annual Report, the CRCAH acknowledged that key issues to be addressed were providing support for researchers and reviewers, and ensuring that the proposals met priorities of industry. Whereas in the past individual researchers had controlled the development of projects and staff had overseen the assessment processes, the programs structure would ensure that the CRCAH took a 'guiding role in the development of research projects to make sure they met the Board's priorities and were developed in a constructive, collaborative way, instead of competitively'.

The CRCAH held industry roundtables to discuss the priorities for research in each program. The roundtables generally involved 20 to 40 representatives from Aboriginal health and relevant government organisations meeting with CRCAH Program Managers and leaders. The CRCAH 2005-2006 Annual Report describes the roundtables as an opportunity where 'Aboriginal health service managers and workers use their direct experience to discuss their most pressing needs to help them provide better services'.

The CRCAH Board ranked the priorities identified at the roundtable according to where they could have the most impact. Program Leaders then translated priorities into research questions. These questions were circulated to the CRCAH community with an invitation for individuals to nominate for the leading, conduct or reviewing of project proposals [36].

Also considered in the third CRCATH monograph mentioned above was the issue of broadening peer review to include social merit as well as scientific merit. The authors found that while funding bodies want both high quality and socially relevant research, performance indicator systems generally are much better at measuring scientific excellence than social impact [25]. In order to address this, the *Practical Solutions for Peer Review *project mentioned above was commissioned to investigate peer review. The findings that 'evidence for the efficacy of peer review is slight' and the discussion of alternative models contributed to the CRCAH's consideration of how to review the proposals [2].

A quality assurance (QA) process was developed in which written reviews assess social relevance (merit review) as well as science (traditional peer review). These reviews were not conducted anonymously. Reviewers and Project Leaders met face to face to 'workshop improvements to the project' [37]. Research proposals were not approved until the CRCAH was confident that the quality was of a high standard. More than one round of reviewing was sometimes necessary. The QA workshops were a two-way learning process in which researchers and non-researchers had an opportunity to listen to each other and strengthen the projects. Involvement in discussions about the research enhanced non-researchers' ability to respond to proposals. Researchers had the opportunity to develop and refine proposals in response to constructive criticism.

The CRCAH stressed to its Project Leaders that its processes were different to the 'traditional' way research is developed, and that Program Managers would be in close communication with Project Leaders during the development and conduct of the research to ensure it met the priorities identified by the Aboriginal health sector [38].

An example of how the facilitated development process was different to the traditional grant-funding round can be seen in the development of Project X. This project was put forward in the grant-funding round in 2004 and did not get funded. In 2005, some proposals that had been unsuccessful in their bid for funding were revisited. Project X was seen to address important issues in both stakeholder consultations and Board priorities. Although in the grant-funding round the topic was seen as important, the methodology and the input from stakeholders were not seen as robust. With the CRCAH's support, the proposal was strengthened by ensuring it included appropriate methods and expertise, and is now a funded research project.

## Discussion

The process of changing how the CRCAH makes decisions on funding research has been a huge task for all involved. It was described by Jenny Brands, the Research and Development Manager, as 'turning the oil tanker' [39]. But feedback to date suggests that the new approach has the support of stakeholders. In late 2006, an unpublished industry survey for the CRCAH Third Year Review found that ' [o]verall, the CRCAH appears to be an effective organization that is on track to achieve its objectives, in a manner that satisfied its partners and research-users'.

More recently, CEO Mick Gooda has given examples of how the CRCAH research is being welcomed by research users. For instance, when presenting the body of work around the Primary Health Care and Health Systems area to senior managers involved in the Commonwealth Department of Health and Ageing, he was able to highlight congruence between the priorities of the Department and the CRCAH. He has also found an overwhelming response from Aboriginal health services and government departments when the CRCAH has sought partners for two projects in support systems and in quality standards which aim to bring about cultural appropriateness and quality services in both mainstream and Aboriginal-run services [40].

One of the main criticisms of the facilitated development process is the claim that it is time-consuming and resource-intensive. The independent panel of the Third Year Review expressed concern that there was a risk of leaving a short time for project implementation and dissemination. However, the CRCAH has found that this process is a comparatively efficient investment of time and resources. Although delays are frustrating, this approach contributes towards strengthening partnerships and producing robust research [35]. The likelihood of meeting priorities is far greater than in the traditional grant-funding round. (An example of traditional grant-funding can be seen in the NHMRC experience of rejecting more than 75 per cent of proposals, sometimes with no feedback to inform their development.)

Discussions of priority setting in the literature generally focus on the institutional level. But the CRCAH's experience is that priorities set by the Board and stakeholders will not necessarily translate into research conducted at a project level. Priority setting might be seen to be a two-stage process: setting the overarching organisational priorities is followed by narrowing these into more specific priorities for funding individual projects. The former usually involves a great deal of consultation but the latter process of ranking projects for allocating funding usually values methodology and track record alongside stakeholder priorities. Proposals that meet priorities but do not meet the scientific criteria are not likely to be funded. But it might be argued that this is a case of throwing out the baby with the bathwater. And in such a process, a lot of time and resources are allocated to developing proposals that are never funded.

In contrast, the CRCAH's processes challenge traditional processes by focusing on developing research questions around identified priorities. Rather than setting priorities and then asking researchers to go away and come back with a research question, the CRCAH brings together industry and researchers to develop the research question as part of the process of developing high-level priorities. Then these research questions are developed into proposals that are subject to a QA (Quality Assurance) process. This QA process focuses on assessing and assisting proposals to reach an appropriate quality rather than ranking them against each other on scientific and social criteria. One of the benefits of this process is that the project teams are supported in the development of high quality proposals that are likely, though not guaranteed, to be funded.

The CRCAH facilitates the development of research that meets priorities through strengthened partnerships and robust methodologies. In transforming research development from an individual to an organisational process, the research is likely to be more useful to stakeholders.

Some researchers have had difficulties with these changes because they are accustomed to an investigator-driven model. At times, they have felt unclear about what was required of them. The demands on the CRCAH to clarify and communicate complex and unusual processes have been huge. Asking researchers to develop projects for a defined topic has also been a challenge. The CRCAH has struggled at times to find Project Leaders with the skills, time and interest to take on an identified project. This has pointed towards a risk that the process might be unbalanced in favour of non-researchers, and has demonstrated the importance of researchers' involvement if projects are to be realised. Lastly, some researchers have struggled with the reduced autonomy in the process, but those that have persevered have found rewards in doing research that is addressing industry priorities and likely to have important outcomes [35].

Lomas has discussed the importance of linkage and exchange in developing useful research in various health services settings [10]. Priority setting processes have been identified to ensure that CRCAH researchers are doing the right research. An additional benefit of these processes is that strong and effective relationships are developed. Despite the limited time available to many of the participants, the industry roundtables and quality assurance workshops have been successful. They have provided spaces for researchers and industry to listen to each other, to develop shared understandings and to build research partnerships. This has created a supportive environment to nurture emerging researchers by exposing them to constructive feedback rather than bruising criticism. These linkage and exchange outcomes reflect the capacity of priority setting processes to produce benefits beyond the identification of research priorities. These processes have also been shown to strengthen the research, to build capacity for research, and to encourage policymakers and practitioners to understand and value evidence. Another important outcome of the CRCAH's processes is to help Aboriginal people have a good experience of research and understand how it can contribute to better health outcomes.

## Conclusion

Priority setting is about making decisions. Key issues faced during priority setting processes include identifying *who *makes these decisions, *who *sets the criteria, and *who *benefits. Historically, researchers have made decisions about what research gets done. Many stakeholder groups, including Aboriginal communities, have begun to demand appropriate consultation on priorities to ensure that research results in benefits to the wider community.

Traditionally, projects have been identified through competitive grant-funding processes. Such processes were found by the CRCAH to be inadequate in meeting priorities: they assessed scientific merit but did not give sufficient weight to stakeholders' concerns. In addition, the processes were found to have outcomes that did not support the CRCAH's objectives of developing research capacity and collaborative partnerships. However, the experience of the CRCAH is that where research priorities are set at an organisational level through stakeholder consultation, there are still risks that these priorities will not be met in the development and conduct of research. A review of priority setting found that traditional processes were ineffective and a new approach was needed. In response, a new model of facilitated project development was implemented. In this model, CRCAH staff were involved in the development of research projects to ensure they reflected stakeholder priorities as well as producing high quality research. It has also been successful in bringing together researchers and research users in the development of projects.

Given the current state of Indigenous health and social issues in Australia, there is an urgent and overwhelming requirement for health research to directly address Indigenous priorities. The CRCAH's experience has demonstrated that a collaborative process is more effective than a competitive one in developing projects that will deliver the benefits that priority setting promises. It has also demonstrated that, by providing meaningful support, a collaborative process can develop the skills of researchers at all levels and produce robust and useful research.

## Competing interests

JM has been funded to work as staff member of the CRC for Aboriginal Health (CRCAH). IA is the Research Director of the CRCAH in a part time and in-kind capacity. KR is a program leader of the CRCAH in a part time and in-kind capacity.

## Authors' contributions

IA and JM developed the concept of the article. JM drafted the article, with comments and additional writing by KR and IA in finalising the article. All authors read and approved the final manuscript.
